# Toxin and Growth Responses of the Neurotoxic Dinoflagellate *Vulcanodinium rugosum* to Varying Temperature and Salinity

**DOI:** 10.3390/toxins8050136

**Published:** 2016-05-05

**Authors:** Eric Abadie, Alexia Muguet, Tom Berteaux, Nicolas Chomérat, Philipp Hess, Emmanuelle Roque D’OrbCastel, Estelle Masseret, Mohamed Laabir

**Affiliations:** 1 Institut Français de Recherche pour l’Exploitation de la Mer (IFREMER), Laboratoire Environnement Ressources du Languedoc-Roussillon, Centre for Marine Biodiversity, Exploitation and Conservation (MARBEC), CS30171 Sète Cedex 03 34200, France; alexia.muguet@hotmail.fr (A.M.); tom.berteaux@ifremer.fr (T.B.); emmanuelle.roque@ifremer.fr (E.R.D.); 2 Institut Français de Recherche pour l’Exploitation de la Mer (IFREMER), Laboratoire Environnement Ressources de Bretagne Occidentale, Place de la Croix, Concarneau 29900, France; nicolas.chomerat@ifremer.fr; 3 Institut Français de Recherche pour l’Exploitation de la Mer (IFREMER), Laboratoire Phycotoxines (DYNECO/PHYC), Rue de l’Ile d’Yeu, BP 21105 Nantes Cedex 3 44311, France; philipp.hess@ifremer.fr; 4 Center for Marine Biodiversity, Exploitation and Conservation (MARBEC), Université de Montpellier (UM), Institut de Recherche pour le Développement (IRD), Ifremer, Centre National de la Recherche Scientifique (CNRS), Place E. Bataillon, CC93, Montpellier Cedex 5 34095, France; estelle.masseret@univ-montp2.fr (E.M.); mohamed.laabir@univ-montp2.fr (M.L.)

**Keywords:** *Vulcanodinium rugosum*, Mediterranean Ingril Lagoon, toxin production, growth conditions, temperature, salinity

## Abstract

*Vulcanodinium rugosum*, a recently described species, produces pinnatoxins. The IFR-VRU-01 strain, isolated from a French Mediterranean lagoon in 2010 and identified as the causative dinoflagellate contaminating mussels in the Ingril Lagoon (French Mediterranean) with pinnatoxin-G, was grown in an enriched natural seawater medium. We tested the effect of temperature and salinity on growth, pinnatoxin-G production and chlorophyll *a* levels of this dinoflagellate. These factors were tested in combinations of five temperatures (15, 20, 25, 30 and 35 °C) and five salinities (20, 25, 30, 35 and 40) at an irradiance of 100 µmol photon m^−2^ s^−1^. *V. rugosum* can grow at temperatures and salinities ranging from 20 °C to 30 °C and 20 to 40, respectively. The optimal combination for growth (0.39 ± 0.11 d^−1^) was a temperature of 25 °C and a salinity of 40. Results suggest that *V. rugosum* is euryhaline and thermophile which could explain why this dinoflagellate develops *in situ* only from June to September. *V. rugosum* growth rate and pinnatoxin-G production were highest at temperatures ranging between 25 and 30 °C. This suggests that the dinoflagellate may give rise to extensive blooms in the coming decades caused by the climate change-related increases in temperature expected in the Mediterranean coasts.

## 1. Introduction

Many dinoflagellate species are responsible for Harmful Algal Blooms (HABs) with a negative impact on economic activity and human health [1]. Results obtained within the frame of the monitoring network, “REPHY” (French phytoplankton and phycotoxins monitoring network), created in 1984, have indicated an increase in the frequency of HABs and their spread along French coastal areas. In French Mediterranean lagoons, *Alexandrium catenella*, which produces paralytic shellfish poisoning toxins, has often been associated with shellfish intoxications and farm closures in Thau lagoon [2,3,4,5], e.g., caused by high cell concentrations *in situ* having reached 14 × 10^6^ cells L^−1^ in 2004. In 2007, mussels collected in the Ingril Lagoon (Mediterranean, France) presented atypical toxicity, *i.e.*, mouse deaths that could not be explained by the presence of known toxins. Mice death symptoms were not due to regulated lipophilic toxins (okadaic acid, dinophysistoxins, yessotoxins, azaspiracids) but were related to neurotoxins [6]. This atypical toxicity had been observed in this lagoon for several years. In 2010, the mouse bioassay was replaced by liquid chromatography coupled with tandem mass spectrometry (LC-MS/MS) which allowed detailed measurement of the lipophilic toxins in shellfish confirming that the above mentioned lipophilic toxins could not be incriminated. The fast acting atypical toxicity corresponded to a toxic imine group, pinnatoxins, which caused the observed death of mussels in the Ingril Lagoon. These neurotoxins, first isolated from *Pinna muricata* in Japan [7,8], have been described as potent shellfish poisons. A new dinoflagellate was found in the Japanese waters in 2010 and described as a pinnatoxin producer [9]. In 2010, a dinoflagellate species was isolated in the Ingril Lagoon and identified as a new species named *Vulcanodinium rugosum* [10]. The species was suspected to be the producer of pinnatoxins and thus responsible for the atypical toxicity observed in mice. Recent studies have clearly shown that pinnatoxin accumulation in shellfish is related to the presence of *V. rugosum in situ* [11,12]. In the Ingril Lagoon, pinnatoxin G (PnTX-G) was found in mussels and clams at high levels ranging from 37 to 459 µg PnTX-G kg^−1^ in mussels and 17 to 95 µg PnTX-G kg^−1^ in clams [6]. These authors also showed that a strain of *V. rugosum*, isolated from the Ingril Lagoon (IFR-VRU-01) when grown in culture, produced PnTX-G (4.7 pg cell^−1^).

The development of HAB species and the resulting blooms are controlled by complex biotic and abiotic factors. Among them, water temperature and salinity are believed to greatly influence the biology and physiology of dinoflagellates and thus their population dynamics. Laboratory and environmental studies have demonstrated the role of water temperature and, to a lesser extent, salinity on the growth of harmful algal species, and therefore on the formation and decline of blooms [2,13,14,15,16,17,18]. Water temperature and salinity fluctuations could impact many physiological processes in HABs, such photosynthesis, toxin production and growth [14,19,20,21,22,23]. Water temperature significantly affects the dynamics of HABs by regulating the formation rate of the dormant stage. Water temperature also affected excystment and thus their ability to inoculate the water column [24,25] as well as the growth of resulting vegetative cells [2,26]. Studies found that salinity was correlated with cell toxin concentrations [2,26,27,28,29,30,31,32,33]. However, most of the studies were performed on planktonic dinoflagellates such as species of the genus *Alexandrium* with only a few studies being carried out on benthic dinoflagellates [34,35]. To our knowledge, no studies have investigated the effects of temperature and salinity on the growth and toxin production of *V. rugosum* a species described as benthic by Nézan & Chomérat [10] and Zeng *et al.* [12]. Data on environmental factors affecting the toxin content in benthic and bentho-pelagic dinoflagellates from various geographic regions and particularly from the Mediterranean Sea are still scarce.

This work aimed at determining the effects of different degrees of salinity and different temperatures tested in combination on the growth and toxin production of *V. rugosum*. The strain tested, IFR-VRU-01 was isolated from Ingril Lagoon in 2010 and grown in nutrient-replete laboratory culture, to obtain the information needed regarding the physiology of this dinoflagellate to better understand its population dynamics in a natural environment.

## 2. Results

### 2.1. Effect of Environmental Factors on Growth

#### 2.1.1. Temperature

**Figure 1:**
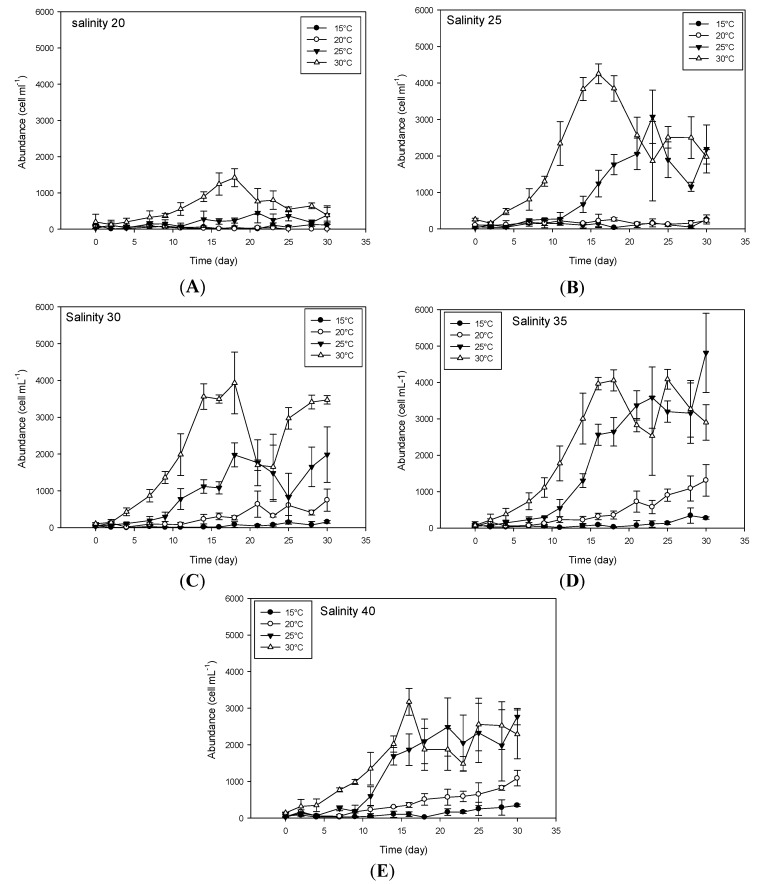
Growth pattern of *Vulcanodinium rugosum* IFR-VRU-01 strain exposed to different temperature and salinity conditions. The curve (**A**) represent the tested temperatures for salinity 20; the curve (**B**) represent the tested temperatures for salinity 25; the curve (**C**) represent the tested temperatures for salinity 30; the curve (**D**) represent the tested temperatures for salinity 35 and the curve (**E**) represent the tested temperatures for salinity 40. Bars represent the standard deviation of the triplicates.

**Figure 2:**
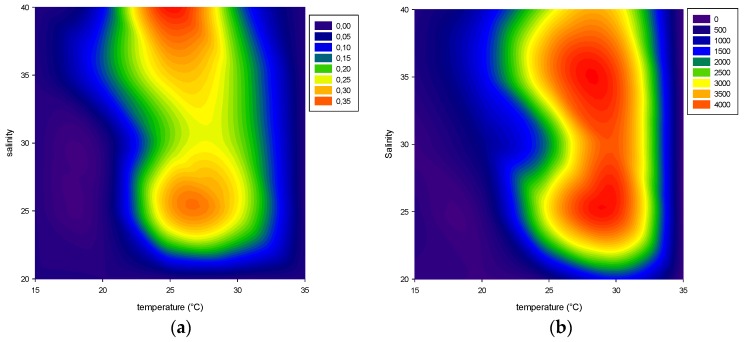
*Vulcanodinium rugosum* IFR-VRU-01 strain growth rate in day^−1^ (**a**) and cell yield in cells mL^−1^ (**b**) in relation to the tested salinity and temperature.

In this study we observed that *V. rugosum* was able to grow at temperatures ranging from 20 to 30 °C, while it was not able to grow at 35 °C, the highest temperature tested (Figure 1 and Figure 2). *V. rugosum* did also not grow at 15 °C irrespective of the salinity of the medium. The dinoflagellate showed the lowest growth rate (0.10 d^−1^) at 20 °C compared to the values obtained at 25 °C and 30 °C. Also the maximum cell density (cell yield) was the lowest (maximum 1311 cells mL^−1^) at 20 °C (Figure 1). The optimal temperatures for *V. rugosum* growth were 25 and 30 °C with a maximum cell density of 3585 cells mL^−1^ and 4252 cells mL^−1^, and a maximum growth rate of 0.39 d^−1^ and 0.2 d^−1^, respectively. The maximum cell density was reached earlier at 30 °C for all the salinities tested.

#### 2.1.2. Salinity

The most favorable salinity conditions ranged between 25 and 40 at temperatures of 25 °C and 30 °C. Under these conditions, growth rate (µ) ranged between 0.21 d^−1^ and 0.39 d^−1^ and cell density between 1952 cells mL^−1^ and 4252 cells mL^−1^ (Figure 1 and Figure 2). At a salinity of 20, the cell growth was only observed at 30 °C (µ = 0.19 d^−1^), but with a low cell yield (1422 cells mL^−1^). We observed a linear correlation between growth rates and salinity at a temperature of 25 °C and salinity between 30 and 40 (*r*^2^ = 0.627, *p* = 0.011, µ = −0.327 + (0.0177 × salinity)).

### 2.2. Effect of Environmental Factors on PnTX-G Production

**Figure 3:**
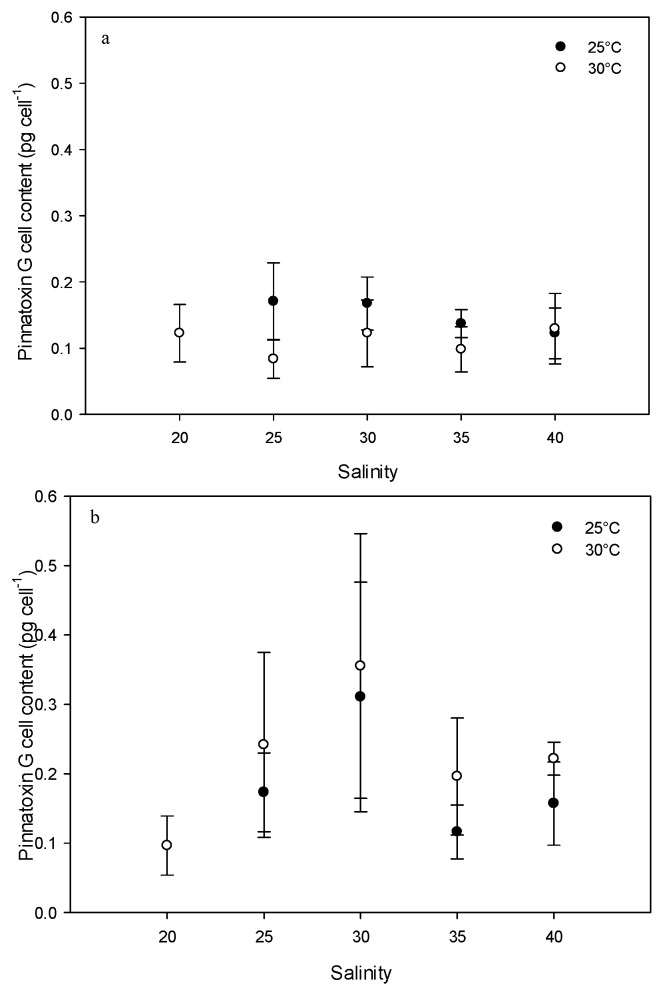
Pinnatoxin G cell content of *Vulcanodinium rugosum* IFR-VRU-01 strain exposed to different salinities and two temperatures. The dots represent PnTX-G concentrations in cell (pg cell^−1^) during the exponential (**a**) and stationary (**b**) phases of growth. Bars represent the standard deviation of the triplicates.

*V. rugosum* did not grow at either 15 or 35 °C. At 20 °C, the exponential and stationary phases were not significantly different. For these reasons, the toxin contents of cells were analyzed for the all salinities tested (only for temperatures of 25 °C and 30 °C). PnTX-G concentrations were dependent on the culture conditions. The lowest concentration (0.08 ± 0.03 pg cell^−1^) was found at a temperature of 30 °C and a salinity of 25 (exponential phase), while the highest concentration (0.36 ± 0.19 pg cell^−1^) was found at a temperature of 30 °C and a salinity of 30 (stationary phase). PnTX-G concentrations at a temperature of 25 °C and during the exponential phase seemed to decrease in relation to salinity (0.17 pg cell^−1^ at a salinity of 25 to 0.12 pg cell^−1^ at a salinity of 40) (Figure 3). In the stationary phase, PnTX-G concentrations appeared to vary as a function of salinity. At the temperature of 25 °C, PnTX-G cell contents increased at a salinity between 25 and 30 (0.13 to 0.31 pg cell^−1^) and decreased at a salinity between 35 and 40 (0.12 and 0.16 pg cell^−1^; Figure 3). At the temperature of 30 °C, similar variations in toxin content were observed as a function of salinity (0.10 to 0.36 pg cell^−1^ at a salinity between 20 and 30; 0.20 and 0.22 pg cell^−1^ at a salinity between 35 and 40). Differences between toxin contents during the different growth phases were more visible at a temperature of 30 °C (salinity > 20 , *p* < 0.05) than at 25 °C (difference only for a salinity of 30, *p* = 0.644). In general, PnTX-G cell contents were lower during the exponential phase than during the stationary phase (*p* < 0.05), particularly at 30 °C (Figure 3).

### 2.3. Effect of Environmental Factors on Chlorophyll Production

**Figure 4:**
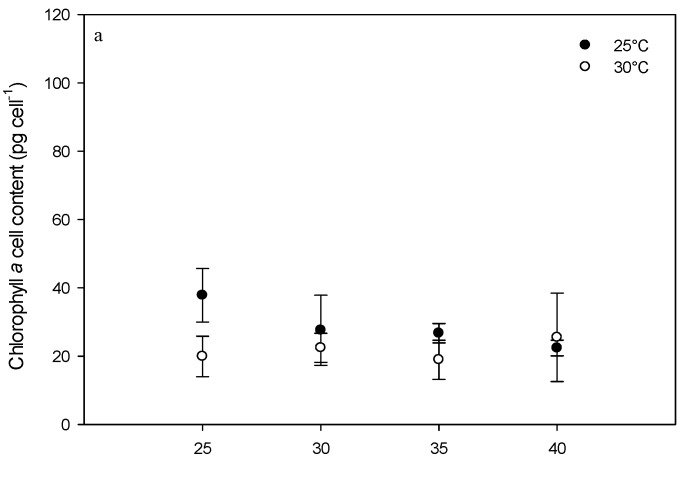

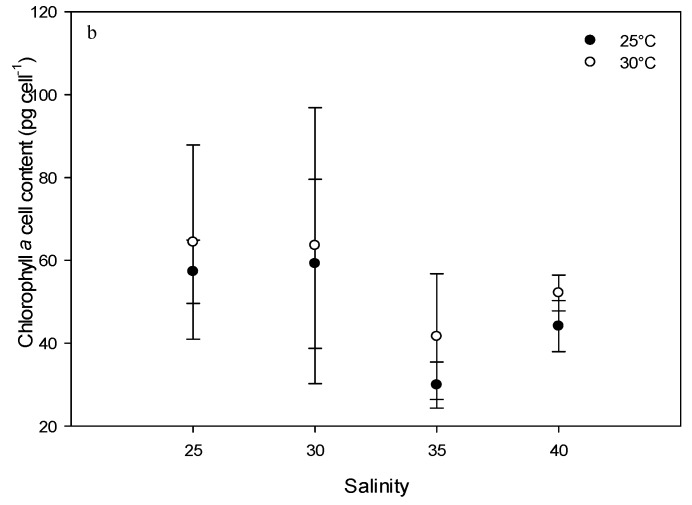
Chlorophyll *a* content in *Vulcanodinium rugosum* IFR-VRU strain cells exposed to different salinities and two temperatures, harvested during the exponential (**a**) and stationary (**b**) phases of growth. The dots represent chlorophyll *a* concentrations in cell (pg cell^−1^). Bars represent the standard deviation of the triplicates.

**Figure 5:**
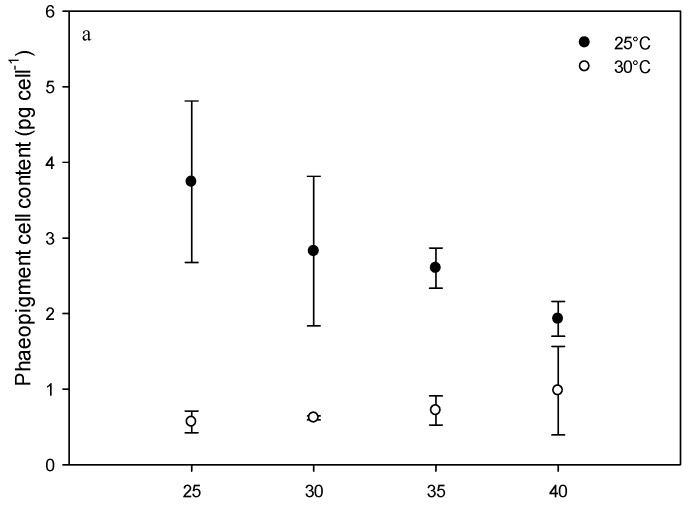

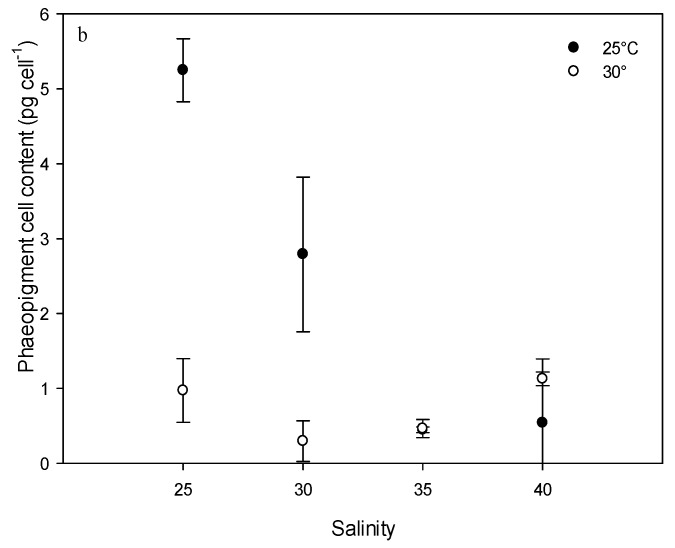
*Vulcanodinium rugosum* phaeopigment *a* contents of cultures grown at 25 and 30 °C and at salinities ranging from 25 to 40 determined during the exponential (**a**) and stationary (**b**) growth phases.

Pigment concentrations were measured only in cultures grown at 25 °C and 30 °C and at all of the tested salinities (except a salinity of 20 at a temperature of 25 °C due to lack of growth) (Figure 4 and Figure 5). During the exponential phase of growth, higher chlorophyll *a* concentrations were observed at 25 °C compared to 30 °C (Figure 4, ANOVA *p* = 0.033; 37.8 ± 7.8 pg cell^−1^ at a salinity of 25 to 22.4 ± 2.3 pg cell^−1^ at a salinity of 40 at a temperature of 25 °C and 19.9 ± 5.9 pg cell^−1^ at a salinity of 25 to 25.5 ± 12.9 pg cell^−1^ at a salinity of 40 at a temperature of 30 °C).

During the stationary phase, no significant difference was found between Chl *a* concentrations at the temperatures tested (*p* = 0.315) (29.9 ± 5.6 pg cell^−1^ at a salinity of 35 to 57.3 ± 7.64 pg cell^−1^ at a salinity of 25 at a temperature of 25 °C and 41.6 ± 15.2 pg cell^−1^ at a salinity of 35 to 64.4 ± 23.4 pg cell^−1^ at a salinity of 25 at a temperature of 30 °C). Chlorophyll *a* production was higher during the stationary phase (*p* < 0.002). When measured during the exponential phase of growth, the phaeopigment concentration was higher for cultures grown at 25 °C compared to those grown at 30 °C (*p* < 0.001) at all the salinities tested. During the stationary phase, these differences were only observed at salinities of 25 and 30 (Figure 5 *p* < 0.016).

## 3. Discussion and Conclusions

### 3.1. Physical and Chemical Parameters Modulate Growth and Toxin Content of Vulcanodinium Rugosum

**Figure 6:**
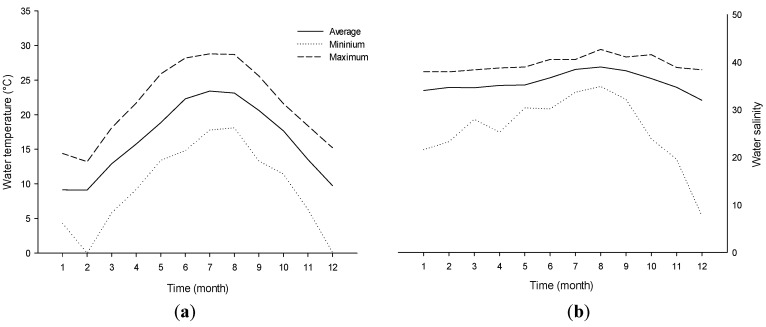
Monthly variation of the water temperature (**a**) and salinity (**b**) of Ingril Lagoon REPHY monitoring 2000–2013.

To our knowledge this is the first time that growth rate and toxin content of *V. rugosum* have been determined over an extensive range of the two main environmental parameters, temperature and salinity tested in combination. In Ingril Lagoon, the rain pattern is characterized by significant inter-annual variability (200–1000 mm year^−1^), and the temperature and salinity also vary widely as a function of season and water depth: for example, they ranged from 0 to 29 °C and from 17 to 42, respectively, between 2000 and 2013 (REPHY monitoring program, Figure 6). These variations are comparatively high, which is related to the low depth of the water column in this lagoon. Indeed, the monthly average temperature in this lagoon was > 20 °C between May and September and the monthly average salinity ranged between 32 and 39 (with very important monthly variations). Our results showed that *V. rugosum* from Ingril Lagoon could not grow at temperatures ≤15 °C and ≥35 °C. At 20 °C, the dinoflagellate did develop, but at a relatively low rate (0.13 d^−1^) and with a long lag phase (18 days) when the salinity was ≥30. *V. rugosum* showed the highest growth rates and maximum cell densities when it was grown at 25 °C (0.21 to 0.39 d^−1^) and 30 °C (0.16 to 0.26 d^−1^), and when salinities ranged from 25 to 40, which suggests that the species is thermophile. As a comparison, *Alexandrium catenella* from the Thau lagoon was found to grow at temperatures and salinities ranging from 15 to 27 °C and from 31 to 40, respectively [21,29,36,37]. The optimal temperature and salinity for *V. rugosum* growth were close to the values reported for other epiphytic or benthopelagic dinoflagellates such as *Gambierdiscus* spp. [34,35] and *Ostreopsis* spp. from Thailand, which showed high growth rates (0.47 to 0.56 d^−1^) at temperatures and salinities ranging between 25 °C and 30 °C and between 25 and 35, respectively [38]. *V. rugosum* from Ingril Lagoon showed growth values lower than those of *A. catenella* isolated from Thau lagoon which reached up to 1.0 d^−1^. The growth rate of *V. rugosum* was closer to those of *Ostreopsis* spp. (0.17 d^−1^ [39] 0.34 d^−1^ [40] and 0.49 d^−1^ [41]) and *Prorocentrum lima* (0.34 d^−1^ [42]). The linear regression between growth rate and salinity observed with *V. rugosum* is usually not described for other dinoflagellates. This relationship has been shown either for both *Gambierdiscus* [34] and *Prorocentrum donghaiense* [23].

Nutrient concentration and ratio, temperature and salinity may all affect cellular toxin content in many dinoflagellate species [21,37,43,44,45,46,47]. Abadie *et al.* [48] showed that PnTX-G content of *V. rugosum* cells was significantly lower with urea as a nitrogen source compared to nitrate- and ammonium-based cultures. The IFR-VRU-01 strain which was isolated in the Ingril Lagoon and used for our laboratory experiments produces only PnTX-G with a minimum and a maximum amounts of 0.08 pg cell^−1^ and 0.36 pg cell^−1^, respectively. Here, *V. rugosum* cell toxin content was lower during exponential phase compared to that observed during stationary phase for the tested temperature (*p* = 0.012). This could be explained by the increase of the energy allocated to toxin synthesis instead of cell division and an increase in cell size often correlated with an increase in cell toxin content. Similarly, Guerrini *et al.* [40] showed that the toxin content in two strains of *O. ovata* was higher during the stationary phase. In contrast, toxin content was higher during the exponential phase in *Alexandrium catenella* and *Pyrodinium bahamense* [26,49].

The cell concentrations of PnTX-G found for the French Mediterranean *V. rugosum* strain (IFR-VRU-01) are particularly low (0.08–0.36 pg cell^−1^) when compared to those previously observed for other strains of the same species developing in Pacific and Indian oceans, *i.e.*, 11.9 pg cell^−1^ for CAWD188 [9] and 87 pg cell^−1^ for CAWD180 [50]. The differences did not seem to be related to culture conditions including temperature and salinity (Table 1) but to the intraspecific variability within this species. Interestingly, in this study, the PnTX-G cell content of the IFR-VRU-01 strain from the Ingril Lagoon was relatively low compared to the toxin content determined for the same strain by Hess *et al.* [6] (4.7 pg cell^−1^) in 2012. The cell content of PnTXs might decrease with culture age. The IFR-VRU-01 strain was isolated in 2010 and maintained in the laboratory through successive culturing which may have led to genetic mutations and alteration of the bacterial flora. Similarly, the dinoflagellate *Alexandrium minutum* had been shown to become less toxic after prolonged culturing [51]. Likewise, another clone of a strain of the paralytic shellfish toxin producer *Alexandrium lusitanicum* (synonym of *Alexandrium minutum* Halim) completely lost its toxicity in culture [52].

### 3.2. Worldwide Distribution of *V. rugosum* and Related Toxins

**Figure 7:**
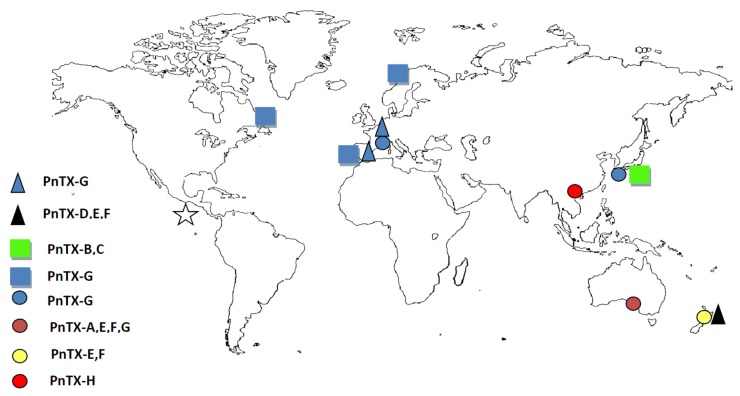
Worldwide distribution of pinnatoxins and/or *Vulcanodinium rugosum* (*V. rugosum*). The symbols presented below are plotted in different colors to indicate which pinnatoxin (PnTX A to H) was found and in which geographic location. Pinnatoxins were detected in *V. rugosum* cells isolated in the studied area (circle), only in shellfish (square), only in SPATT (passive sampling device) (triangle). *V. rugosum* cells were identified in the studied area but no toxin analysis was performed (star).

**Figure 8:**
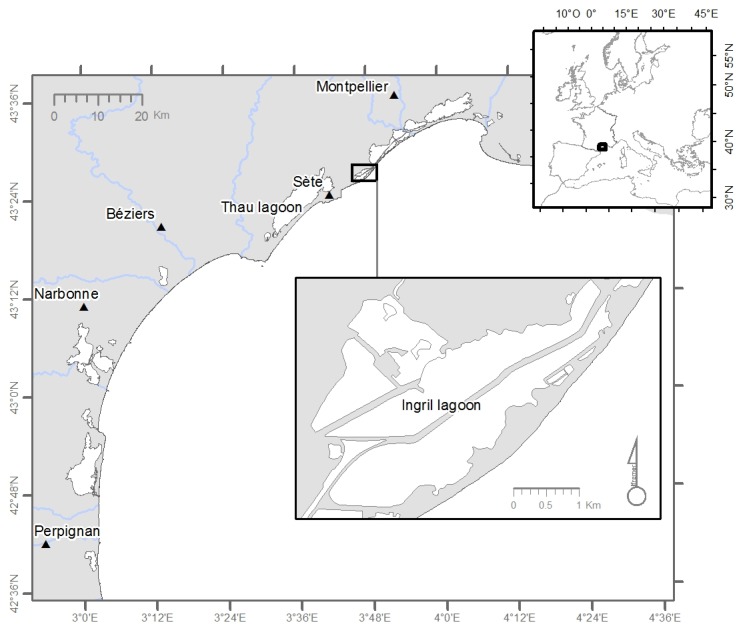
Ingril Lagoon (French Mediterranean coast).

Recent works show that *V. rugosum* is widely distributed around the world suggesting that it could be considered as a cosmopolitan species [6,9,12,54,57,58] (Figure 7, Table 1). Natural or human-assisted dispersion (ballast waters, shellfish relocation ...) or the development of endemic populations could explain this geographic distribution [59]. Further studies have to focus on the phylogeny of isolated strains of *V. rugosum* to better understand the geographic distribution of this probably expanding harmful dinoflagellate. Concerning the toxins produced by the dinoflagellate, data so far show important toxin diversity varying as a function of the strains colonizing each of the oceans. In the Pacific, PnTX-E and F dominated in the strains originating from New Zealand (CAWD163–178), whereas PnTX-G was the only toxin detected in Japanese strains (CAWD188, 190), and the Chinese strain CAWD198 produces only PnTX-H. The Australian strains produce PnTX-G and H (CAWD180, 183) (Table 1 and references therein). Selwood, *et al.* [55] discovered the presence of portimine, a new cyclic imine, in a *V. rugosum* strain isolated from Northland, New Zealand. Regarding isolates from the Southern Ocean, pinnatoxins A, E, F and G were found in two strains (CAWD180 and CAWD183) isolated from Franklin Harbour, Australia. In contrast, the strain IFR-VRU-01 from Mediterranean waters has been shown to produce only PnTX-G [6]. As suggested for *Alexandrium* [60], the production of toxins by *V. rugosum* may be genetically driven for each clonal strain. In addition, pinnatoxins were found in passive sampling devices (SPATT) and shellfish in various marine ecosystems, despite *V. rugosum* cells not being reported in those same studies. In Mediterranean waters (Catalonia, Spain), PnTX-G was detected by solid phase adsorption toxin tracking (SPATT) [61]. This passive sampling technique revealed the presence of PnTX-E and F and traces of PnTX-A, D and G in New Zealand waters [62]. PnTX-B and C were detected in the Okinawan bivalve *Pinna muricata* [63]. PnTX-G was also detected in different shellfish species from the Eastern Canadian coast and Norway [64,65]. On the other hand, *V. rugosum* was identified in Mexican waters but no toxin analyses were performed [57]. Interestingly, summarized data relating to toxin analyses of *V. rugosum* cultures maintained in different laboratories (Table 1, Figure 8) showed that PnTX-G was the main pinnatoxin form in Mediterranean and Atlantic waters. Asian and Pacific waters are characterized by higher pinnatoxin diversity as all the PnTX analogues were detected in the related ecosystems (PnTX-A, E, F, G and H). PnTX B and C were found only in the Okinawan (Japan) bivalve *Pinna muricata*. This suggests that *V. rugosum* may well be an introduced species in the Mediterranean and Atlantic seas. Surprisingly, these PnTXs (B and C) were not found in mussels and clams from Ingril Lagoon (unpublished data) whereas PnTX-A was present in these mollusks at non-quantifiable traces [6]. Overall, these data matched [66] suggestions about the biotransformation of algal metabolites PnTXs-E, -F and -G into shellfish metabolites PnTXs-A, -B, -C and -D. Quantification of PnTXs by LC-MS/MS revealed significant variability in *V. rugosum* cell contents (PnTX-E: 0.4–10 pg cell^−1^, PnTX-F: 2.3–41 pg cell^−1^, PnTX-G: 0.14–87 pg cell^−1^) which may be directly related to the strain or to culture conditions. Pinnatoxins exhibit fast-acting toxicity when injected intraperitoneally (i.p.) into mice [58]. The high intrinsic toxicity of PnTXs is indicated by low LD_50_ values following intraperitoneal injection in mice. LD_50_ values varied substantially as a function of the *V. rugosum* strain and the intracellular toxin injected (CAWD167–PnTX-E and F: 1.33 mg kg^−1^, CAW183–PnTX-G: 42.7–48 µg kg^−1^, CAWD198–PnTX-H: 67 µg kg^−1^) (Table 1).

### 3.3. Conclusions

Laboratory data on the ecophysiology of *V. rugosum* are coherent with the fact that this dinoflagellate typically develops during summer time in the Mediterranean.

In 2013, the cell density of *V. rugosum* was found to be at its highest in the summer, between June and September (Abadie *et al.* [67] unpublished data), which confirmed the growth results from our laboratory experiments. PnTX-G concentrations were high in mussels (*Mytilus galloprovincialis*) between June and September 2013. *V. rugosum* has been shown in this study to be a euryhaline dinoflagellate which suggests that this harmful species could spread to other Mediterranean lagoons and confined water systems.

Some studies suggest that there may be relationships between shellfish toxicity and large-scale patterns of climate variability. For example, there was an attempt to explain the increase of *Alexandrium* blooming in the Puget Sound by El Niño which increased the temperature of the water column and produced stratification more suitable to dinoflagellates [68]. Moore *et al.* [69] suggested that aspects of the local climate (daily to seasonal timescales) such as air temperature and stream flow could be more important in determining oceanographic variability in the Puget Sound than large-scale climate variations such as the El Niño Southern Oscillation (=ENSO, *i.e.*, inter-annual to inter-decadal timescales). However, no significant correlations were identified [69] for *Alexandrium* in the Puget Sound marine system. It is undeniable that marine ecosystems are facing a global temperature increase [70,71,72,73]. For example, 2014 was ranked as the warmest year since the beginning of weather records in France. Southwestern Mediterranean lagoons are characterized by the relatively low depth of their water columns (e.g., Ingril Lagoon: 0–1.7 m) which means that water temperature could increase more rapidly and stay warm for longer periods following any warming. Our data showed that *V. rugosum* is thermophile like other benthic dinoflagellates and its growth is mainly driven by temperature which suggests that this dinoflagellate may be responsible for blooms in the coming decades caused by the climate change-related expected increase in temperature of the Mediterranean area.

## 4. Experimental Section

### 4.1. Vulcanoidinum rugosum: Origin and Culture

The IFR-VRU-01 strain of *V. rugosum* was isolated from the Ingril Lagoon (French Mediterranean coast) in 2010 by Nézan & *Chomérat* [10] (Figure 8). IFR-VRU-01 was shown to produce PnTX-G [6]. The non-axenic culture of *V. rugosum* was maintained at a temperature of 25 °C, a salinity of 35 and an irradiance of 100 µmol photon m^−^^2^ s^−1^ in an Enriched Natural Sea Water medium (ENSW) [74] in the MARBEC laboratory (Montpellier, France).

As described by Rhodes *et al.* [11], the life cycle of *V. rugosum* is characterized by typical, motile vegetative cells and unornamented, non-motile cells (30–32 mm diameter). For each experiment testing the effect of temperature and salinity, the flasks were inoculated only with healthy motile cells. Our microscopic observations clearly showed that the cultures were dominated largely by motile cells (>90%) until the stationary phase when non motile cells appeared and settled against the flask walls. Growth rate was calculated for the exponential phase based only on cell counts of motile cells. PnTX-G and Chlorophyll *a* (Chl *a*) levels were measured during the exponential phase and at the beginning of the stationary phase when motile cells still dominated.

### 4.2. Experimental Conditions

Seventy-five 250 mL-flasks (Cellstar^®^ Cell Culture Flasks, Greiner Bio-One, Courtaboeuf, France) were inoculated. The flasks were filled with ENSW medium adjusted at five different salinities (20, 25, 30, 35 and 40). Each flask was inoculated at initial concentration of 400 cells mL^−1^ in a total volume of 200 mL. For each salinity, three flasks (replicates) were placed in four incubators (SANYO Incubator, Firlabo, Panasonic Healthcare Company, Etten-Leur, the Netherlands, Meditest 600/1300, Panasonic MIR-554, Panasonic Healthcare Company, Etten-Leur, the Netherlands, and one temparated-controled room) at different temperatures (15, 20, 25, 30 and 35 °C). The test at 35 °C was separately conducted after the first four tests. Cultures were exposed to an irradiance of 100 µmol photon m^−^^2^ s^−1^ using cool white fluorescent lights and a 12:12 h light:dark cycle. Temperature of culture chambers were monitored throughout the experiment by a temperature probe (Proges Plus 22 L). Light irradiance was controlled using a PAR sensor (PQS 1 KIPP ZONEN, Delft, The Netherlands). The salinity of each medium was checked during the experiment with a WTW LF 197 probe (WTW Wissenschaftlich-Technische Werkstätten GmbH, Weilheim, Germany).

### 4.3. Cell Concentrations and Growth Rate Measurements

The cell density of each flask was monitored every two days for thirty days. After gentle homogenization to avoid any stress to the cultures, 1350 μL aliquots of culture were withdrawn sterilely with a micropipette and counted with a Nageotte cell (counting chamber) under an inverted light microscope (Zeiss Axiovert 25, Carl Zeiss SAS, Marly Le Roy, France). In accordance with Guillard [75], the growth rate (μ; expressed in day^−1^) was calculated from the slope of a linear regression line over the entire exponential phase of growth by the least squares fit of the straight line to the data after logarithmic transformation; μ = Ln (*N*_1_) – Ln (*N*_0_) /*T*_1_ – *T*_0_ in units of day^−1^ where *N*_1_ and *N*_0_ were cell densities at times *T*_1_ and *T*_0_, respectively, during the linear portion of exponential growth phase.

### 4.4. Chlorophyll a Analysis

Chlorophyll *a* concentrations were determined for each flask when the cultures were in the exponential and stationary phases. For Chl *a* measurements, culture (15 mL) was withdrawn and filtered on a 25 mm-diameter GF/F Whatman filter at a low pressure (<100 mbar) to avoid cell lysis. Filters were placed in 12 mL-polypropylene-tubes and stored at −20 °C. Aqueous acetone (5 mL, acetone:water 9:1) was used for extraction and filters were decomposed using an ultrasonic rod (Vibra cell™ 7518, Sonics & Materials, Inc., Newtown, CT, USA, 130 Watt—50 kHz). The mix was centrifuged at 2750× *g* and 4 °C for 30 min. The supernatants were analyzed by spectrofluorimetry (Perkin Elmer, PerkinElmer Inc., Waltham, MA, USA, LS50B) using the method described by Neuveux [76]. Pigment concentration was expressed in pg cell^−1^. To calculate the toxin concentration per cell, the cell density of the chemically analyzed sample was used.

### 4.5. Pinnatoxin G Measurement

**Figure 9:**
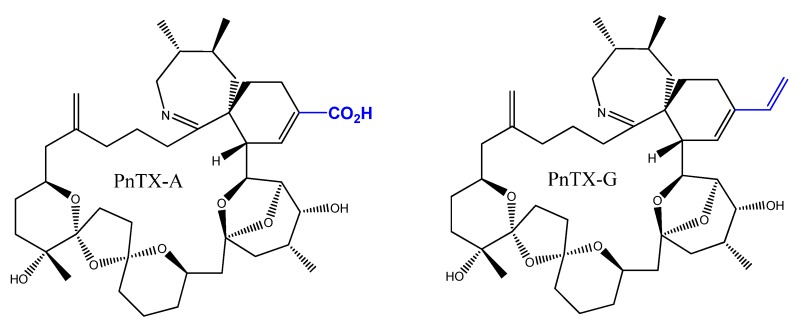
Chemical structure of pinnatoxin A and G.

For the toxin analyses, we took a 10 mL sample from each culture during the exponential and stationary growth phases. The samples were centrifuged (3000× *g*, 15 min, 4 °C) and the supernatants removed carefully. Methanol (100%, 2 mL) was added to the remaining pellet and the samples stored at −20 °C until toxin extraction [54]. Extraction of the pinnatoxins (Figure 9) was carried using three consecutive sonication steps lasting 10 min each, followed by filtration of the extracts over a 0.2 µm membrane (Whatman Mini-UniPrepTM, GE Healthcare Europe GmbH, Velizy-Villacoublay, France). The filtered extracts were stored at −24 °C until quantification. Quantification of PnTX-G was carried out using liquid chromatography coupled to tandem mass spectrometry (LC-MS/MS), using external calibrants over the range of 0.25 to 5 ng mL^−1^. A C8 column (Phenomenex, Phenomenex Inc., Torrance, CA, USA) was used at 25 °C for analysis (injection volume 5 µL). The analysis was conducted at a flow rate of 0.8 mL min^−1^ [6]. The limit of detection (LD) and the limit of quantification (LQ) values were 0.08 and 0.27 ng mL^−1^, respectively.

### 4.6. Statistics

For each set of data, differences between means were analyzed by Student’s t-test or the ANOVA test (Sigmaplot 12.5, Systat Software Inc., San Jose, CA, USA). The appropriate statistical test was conducted after the verification of the normality of the data (Shapiro–Wilk). If the test of the normality failed, a Kruskal–Wallis One Way Analysis of Variance on Ranks (non parametric test) was performed.

Values were expressed for each condition as the mean (m) of the triplicate and Standard.

## Figures and Tables

**Table 1:** Summary of *Vulcanodinium rugosum* presence in various marine ecosystems, culture conditions, detected toxins and toxicity are specified when available.

Strain and Studied Area	Temperature (°C)	Salinity	Irradiance (µmol photons m^−2^ s^−1^)	Culture Medium	Detected Toxins (Amount in pg cell^−1^)	Toxicity (LD 50 mice)	Reference
Mediterranean waters							
IFR-VRU-01(Ingril Lagoon France)	25–30	30–35	100	ENSW	PnTX-G (0.14–0.36 ) Portimine		This study, Abadie *et al.* (2015) [48]
IFR-VRU-01(Ingril Lagoon France)	18	38	200	L1	PnTX-G (4.7)		Hess *et al.* (2013) [6]
** (Ebre Delta Spain)	**	**	**		**		Satta *et al.* (2013) [53]
Pacific waters							
CAWD163 (Rangaunu Harbour NZ)	25	**	70–100	K	PnTX-E (0.8) PnTX-F (5.1)		Rhodes *et al.* (2010) [54]
CAWD166 (Rangaunu Harbour NZ)	25	**	70–100	K	PnTX-E (3.7) PnTX-F (20.1)		Rhodes *et al.* (2010) [54]
CAWD167 (Rangaunu Harbour NZ)	25	**	70–100	K	PnTX-E (1.4) PnTX-F (8.4)		Rhodes *et al.* (2010) [54]
CAWD168 (Rangaunu Harbour NZ)	25	**	70–100	K	PnTX-E (0.8) PnTX-F (4.6)	IP 1.33 mg/kg gavage 2.33 mg/kg	Rhodes *et al.* (2010) [54]
CAWD170 (Rangaunu Harbour NZ)	25	**	70–100	K	PnTX-E (2.4) PnTX-F (13.6)		Rhodes *et al.* (2010) [54]
CAWD171 (Rangaunu Harbour NZ)	25	**	70–100	K	PnTX-E (0.5) PnTX-F (3.5)		Rhodes *et al.* (2010) [54]
CAWD178 (Rangaunu Harbour NZ)	25	**	70–100	K	PnTX-E (0.4) PnTX-F (2.3)		Rhodes *et al.* (2010) [54]
** (Rangaunu Harbour NZ)	*	**	*	K	Portimine	IP: 1570 µg/kg	Selwood *et al.* (2013) [55]
CAWD188 (Ishigakijima Island Okinawa Japan)	*	**	**	K	PnTX-G (11.9)		Smith *et al.* (2011) [9]
CAWD190 (Ishigakijima Island Okinawa Japan)	*	**	**	K	PnTX-G (15)		Smith *et al.* (2011) [9]
G65 (South China Sea)	20	**	90	f2	new PnTX (20)		Zeng *et al.* (2012) [12]
CAWD198 (South China Sea)	25	**	100	K	PnTX-H	IP 67 µg/kg gavage 163 µg/kg	Selwood *et al.* (2014) [56]
** (Lazaro Cardenas Michoacan Mexico)	18–20	**	90–167	L1SE	**		Hernandez-Becerril *et al.* (2013) [57]
Indian Ocean							
CAWD180 (Franklin Harbour Australia)	25			K	PnTX-G (87) PnTX-E (10) PnTX-F (41) PnTX-A (1.3)		Rhodes *et al.* (2011) [50]
CAWD180 (Franklin Harbour Australia)	25			K	PnTX-G (13)		Rhodes *et al.* (2011) [50]
CAWD183 (Franklin Harbour Australia)	25	**	**	K	PnTX-G	IP Fed: 48.0 µg/kg IP Fasted: 42.7 µg/kg	Munday *et al.* (2012) [58]
